# Sulphur Copolymers with Pyrrole Compounds as Crosslinking Agents of Elastomer Composites for High-Performance Tyres

**DOI:** 10.3390/polym16192802

**Published:** 2024-10-03

**Authors:** Simone Naddeo, Vincenzina Barbera, Maurizio Galimberti

**Affiliations:** Department of Chemistry, Materials and Chemical Engineering “G. Natta”, Politecnico di Milano, Via Mancinelli 7, 20131 Milano, Italy; simone.naddeo@polimi.it (S.N.); vincenzina.barbera@polimi.it (V.B.)

**Keywords:** inverse vulcanization, sulphur copolymers, rubber compounds

## Abstract

Driving a car at extreme speeds, road holding, and sustainability do not go together well. Formula 1 racing is exciting but is not an example of sustainability. The aim of this work was to use materials, suitable for the treads of high-performance racing tyres, that can favour both high performance and sustainability. In particular, the objective was to achieve high dynamic rigidity at high temperatures (>100 °C) and a stable crosslinking network. A copolymer from an industrial waste such as sulphur and a comonomer from a circular biosourced material were used as the crosslinking agent of an elastomer composite based on poly(styrene-co-butadiene) from solution anionic polymerization and a carbon black with a high surface area. The biosourced circular material was 1,6-*bis*(2,5-dimethyl-1*H*-pyrrol-1-yl)hexane (HMDP), the di-pyrrole derivative of hexamethylenediamine. Two poly(S-co-HMDP) copolymers, with different S/HMDP ratios (6 and 8.9, Copolymer 1 and Copolymer 2) were carefully characterized by means of ^1^H-, ^13^C-, 2D^1^H-^1^H-COSY and 2D ^1^H-^13^C HSQC NMR. The comparison of the spectra highlighted the substitution with sulphur of the β-position of the pyrrole ring: mono-substitution largely prevailed in Copolymer 1 and also bi-substitution in Copolymer 2. The copolymers were used as additives in the vulcanization system. Compared with a reference composite, they allowed us to achieve more efficient vulcanization, a higher density of the crosslinking network, higher dynamic rigidity, better ultimate tensile properties, and better stability of the crosslinking network at high temperatures. Compared with a traditional oil-based crosslinking agent for elastomer composites with high rigidity and a stable structure at high temperatures, such as the perthiocarbamate 6-((dibenzylcarbamothioyl)disulfaneyl)hexyl 1,3-diphenylpropane-2-sulfinodithioate, the poly(S-co-HMDP) copolymers led to higher dynamic rigidity and better ultimate tensile properties. These improvements occurring simultaneously are definitely unusual. This work paves the way for the upcycling of circular materials in a large-scale application such as in tyres.

## 1. Introduction

The key feature of elastomers is the entropic elasticity. The elastomers chains, above their glass transition temperature, can be repeatedly stretched to at least twice their original length and recover immediately and forcibly their initial size and shape, upon removing any stress on them [1,2]. To achieve this behaviour, elastomer chains must be linked together. As a matter of fact, the most important unsaturated and saturated elastomers, poly(1,4-cis-isoprene) from natural sources (natural rubber, NR) [3] and poly(ethene-co-propene) (EPR) from insertion polymerization [4], respectively, can have entropic elasticity also in the uncrosslinked state. Both NR [5,6,7] and EPR [8,9,10] have the ability to rapidly crystallize under strain, and the crystallites act as physical crosslinks. The strain-induced crystallization of NR was attributed to the high stereoregularity of the polymer chains [3,11,12,13] and to the small melting entropy [14,15]. More recently, the NR chains have been shown to be linked together, through their chain ends, which are dimethylallyl groups modified with proteins and with phospholipids containing fatty acid esters [16,17,18,19,20,21]. The ionic and hydrogen bonds formed by these polar groups are responsible for the crosslinking and the saturated fatty acids that act as nucleating agents [22,23]. Natural rubber samples from *Hevea Brasiliensis*, dandelion, and guayule all exhibit entropic elasticity in their pristine state [24,25]. Commercially available EPR does not show entropic elasticity in the uncured state. The strain-induced crystallization of EPR was reported in the patent and scientific literature for samples having ethene sequences that are able to crystallize under strain but amorphous at rest and propene sequences whose isotacticity does not disturb the crystallization [8,9,10].

**Figure 1:**
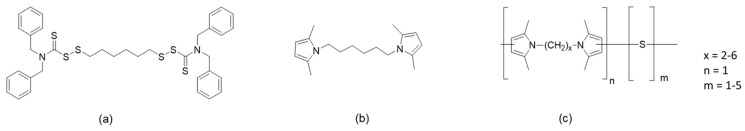
The chemical structure of (**a**) 6-((dibenzylcarbamothioyl)disulfaneyl)hexyl 1,3-diphenylpropane-2-sulfinodithioate (DBTH), (**b**) 1,6-*bis*(2,5-dimethyl-1*H*-pyrrol-1-yl)hexane (HMDP), and (**c**) poly(S-co-HMDP).

It is indeed highly desirable to have elastomers with entropic elasticity in the uncured state. However, to achieve the performance required by their demanding applications, elastomer chains must be chemically crosslinked. Crosslinking dates back to Mesoamericans [26]. Nowadays, many different systems are used to promote the crosslinking of the elastomer chains [27]: they are based on peroxides, phenolic resins, metal oxides, silanes, quinones, maleimides, and particularly, sulphur. The sulphur-based systems contain other chemicals [28]. Indeed, the active sulphurating species must be formed, and to do that, activators and primary and secondary accelerators must be used. The activators are oxides or carboxylates of divalent metals, such as ZnO, zinc stearates, and zinc octanoates. Primary accelerators are sulphenamides, thiazoles, and carbamates. Vulcanization leads to the formation of sulphur bridges between the polymer chains, made by one, two, or more sulphur atoms. The properties of the elastomer composites depend on the length of the sulphur bridges: higher elasticity is achieved with short bridges, at the expense of the fatigue resistance. Hence, a longer service life is achieved with longer bridges. The sulphur bridges experience a dynamic equilibrium: they evolve towards more stable but shorter sequences. Moreover, the chemical bond established by sulphur with the polymer chains is reversible [29,30,31]. It would be highly desirable to have a crosslinking network with a constant structure all over the whole service life of the rubber compound, particularly when it is applied to tyres. This need is particularly relevant in the case of high-performance sport tyres. The objective to have a crosslinking network with higher stability was achieved by adding further ingredients to the vulcanization system, able to establish permanent chemical bonds with the elastomer chains. A typical chemical substance is a perthiocarbamate, 1,6-*bis*(N,N’-dibenzylthiocarbamoyldithio)-hexane (DBTH) [32,33,34], whose chemical structure is shown in Figure 1.

During the sulphur-based vulcanization of an unsaturated elastomer, the cleavage of the sulphur–sulphur bonds releases the amines, which act as accelerators, and the dithioalkane reacts with the elastomer chains, forming bridges more stable than the polysulphidic ones [32]. DBTH is not efficient when it is used as the only vulcanizer [33], but in the presence of sulphur and a sulphenamide [32,33,34], the same crosslink density as the reference compound (without DBTH) and excellent retention of the physical properties were obtained, with the dynamic behaviour remaining almost at the original level even after severe cure conditions [32]. DBTH leads to a higher modulus and lower hysteresis of the composites [34]. However, DBTH presents some technical drawbacks. Its behaviour in vulcanization depends on the formulation of the vulcanization system, and the length of the bridge cannot be tuned. Moreover, it is an oil-based chemical, which cannot be recycled. 

This work had the objective of enhancing the dynamic rigidity and the stability of the crosslinking network of elastomer composites suitable for high-performance tyre treads. This objective was pursued by using an innovative crosslinking agent that could be completely circular and biobased. A sulphur copolymer with a di-pyrrole compound was used as an ingredient of a traditional vulcanization system, based on sulphur and a sulphenamide. Sulphur is largely available, in millions of tons, from oil desulphuration [35]. It is unexpensive and not toxic, but it is flammable, and there is a need to find large-scale applications to remove the accumulated stockpiles. The di-pyrrole compound was 1,6-*bis*(2,5-dimethyl-1H-pyrrol-1-yl)hexane (hexamethylenediaminopyrrole, HMDP), whose chemical structure is shown in Figure 1, the di-pyrrole derivative of hexamethylenediamine, which could become available from the hydrolysis of end-of-life poly(amide-6,6). The di-pyrrole compound was prepared through the reaction of the diamine with 2,5-hexanedione. The authors reported on the synthesis of 2,5-hexanedione from dimethylfuran [36]. The sulphur copolymer with HMDP, poly(S-co-HMDP), was prepared through the reaction of sulphur and HMDP in the typical experimental conditions of the so-called inverse vulcanization [35,37,38,39,40,41,42,43,44,45,46], which means by simply adding HMDP to molten sulphur. The chemical structure of the copolymer is shown in Figure 1. The synthesis and characterization of poly(S-co-HMDP) has been recently reported elsewhere [47]. Two copolymer samples were used in this work, with different amounts of sulphur. The comparative characterization of the two samples is presented. The elastomer composite had a typical formulation for a high-performance tyre tread: it was based on poly(styrene-co-butadiene) from solution anionic polymerization (SBR) with a high styrene content and a carbon black with a surface area much higher than a standard ASTM N100 carbon black such as CRX1391. The poly(S-co-HMDP) copolymer was used as an ingredient of the vulcanization system, and its behaviour was compared to that of DBTH. HMDP was used as well in its pristine state. In the following text, DBTH, HMDP, and poly(S-co-HMDP) are referred to as “crosslinking agents”. The characterization of the composites was carried out by determining the curing kinetics, the dynamic mechanical properties in the shear and the axial modes, and the tensile properties through quasi-static measurements. The structure and the robustness of the crosslinking network were investigated by means of swelling measurements and by performing curing tests at a high temperature (200 °C) for a prolonged time. The one in tyre treads is really a large-scale application: the tyre market is expected to reach 2.7 billion units in the forthcoming year [48]. 

## 2. Experimental Section

### 2.1. Materials

*Synthesis of HMDP as the pyrrole compound (PyC):* 1,6-*bis*(2,5-dimethyl-1H-pyrrol-1-yl)hexane (HMDP) was synthesized according to the literature [47], and 2,5-hexanedione and hexamethylenediamine from Sigma Aldrich were used as the substrates.

*Synthesis of poly(S-co-PyC)copolymers:* HMDP was used as the novel comonomer for the preparation of sulphur-based copolymers. Orthorhombic sulphur, purchased from ZOLFINDUSTRIA Pavia, Italy, was used as the second comonomer. Poly(S-co-HMDP) was synthesized according to the literature [47]. 

*Preparation of elastomer composites.* The solution of Styrene–Butadiene Rubber (SBR) was HP755B and was provided by JSR. The chemical composition of the polymeric matrix was the following: styrene 39.5% wt, vinyl groups 38.2% wt, and extension oil (TDAE, Treated Distillate Aromatic Extract) 37.5% wt. The reinforcing filler used was CRX 1391, purchased from Cabot Corporation (Boston, MA, USA), with a surface area of 1391 m^2^/g. Stearic acid and Zinc salt were provided from Oleo s.r.l. N-(1,3-dimethylbutyl)-N’-phenyl-p-phenylenediamine (6PPD) was used as the typical antioxidant and was purchased from Eastman, Milano, Italy. Zinc Oxide was provided from Lanxess, with a 20% wt of polymeric agent as the dispersing agent. Hansen and Rosenthal provided the oil (Vivatec, Iserlohn, Germany). Sulphur was the same as was used for the preparation of the copolymers. Extension oil (Treated Distillate Aromatic Extract) was used as the processing oil and was purchased from Klaus Dahleke KG, Hamburg, Germany. The Kristalex^TM^ 5140 Hydrocarbon Resin was from Eastman, Kingsport, TN, USA. Accelerating agents like thiuram and Dibenzothiazyl disulphide (MBTS), 80% in weight, were provided by Shandong Yanggu Huatai Chemical Co., Ltd, Yanggu, China. DBTH (Vulkuren, Lanxess, Vienna, Austria) was used as the commercial crosslinking agent and was from Lanxess Deutschland GmbH, Cologne, Germany.

### 2.2. Synthesis of 1,6-Bis(2,5-Dimethyl-1H-Pyrrol-1-yl)Hexane (HMDP)

The synthesis of HMDP was the same as was reported in reference [47]. In brief, hexamethylenediamine (36.6 mmol, 4.27 g) and water (10 mL) were sequentially added to a two-necked round-bottom flask. The mixture was stirred at 37 °C for 30 min, until a homogeneous solution was obtained. Then, droppwised 2,5-hexanedione (73.25 mmol, 8.62 mL) was added to the solution. The obtained reaction mixture was heated to 120 °C and stirred for three hours. Finally, the obtained HMDP was cooled down to room temperature and then washed with distilled water to remove impurity traces. Filtration on a filter paper of the obtained HMDP was performed and dried at 1 atm pressure. A pale-white solid was obtained. HMDP was obtained in 93% of yield, 9.1 g, with an Atom Efficiency of 73%. The calculated E factor was estimated to be 0.46, considering only the organic compounds and neglecting the water used during the washing. The characterization of HMDP was performed by means of NMR spectroscopy and GC-Mass and ESI-Mass analyses.

^1^H-NMR (CDCl_3_), δ (ppm): 5.75 (s, 4H), 3.73–3.70 (m, 4H), 2.21 (s, 12H), 1.67–1.59 (m, 4H), 1.43–1.34 (m, 4H).

^13^C-NMR (CDCl_3_), δ (ppm): 127.28, 105.12, 43.60, 31.06, 26.84, 12.57.

GC-Mass: retention time = 24.20 min; molecular peak = 272 *m*/*z*.

ESI-Mass: [M + H]^+^: Calculated exact mass = 273.22 *m*/*z*; found exact mass = 273.3 *m*/*z*.

### 2.3. Preparation of Poly(S-co-PyC) Copolymers

The syntheses reported below were performed several times, to check the reproducibility of the process and to have samples suitable to reproduce the results of the characterization. The samples used as crosslinking agents were prepared in the work described in a previous publication [47].

#### Synthesis of Poly(S-co-HMDP) Polymers

Sulphur was introduced and heated at 185 °C in a round-bottom flask for ten minutes until a viscous red liquid was obtained. Then, HMDP was added at 185 °C, and the reaction mixture was stirred for an additional ten minutes at 185 °C. The obtained crude product was then cooled down to room temperature. Poly(S-co-HMDP) copolymers were obtained as dark brown solids. 

Poly(Sulphur-co-HMDP) copolymer with the molar ratio S/HMDP = 6 was obtained by using 1.40 g of sulphur (43.67 mmol) and 2.0 g of HMDP (7.34 mmol). 

Poly(Sulphur-co-HMDP) copolymer with the molar ratio S/HMDP = 8.9 was synthesized with 2.10 g of sulphur (65.50 mmol) and 2.0 g of HMDP (7.34 mmol). 

As commented below in the text, residual sulphur and HMDP were not observed at the end of the reactions.

### 2.4. Preparation of Elastomer Composites 

The recipes of the elastomer composites are reported in Table 1.

The same molar amount of sulphur and crosslinking agents were used in all the elastomer composites. The composites were prepared in two steps: (i) non-productive mixing: the preparation of a masterbatch; (ii) productive mixing: the addition of the vulcanization agents. 

#### 2.4.1. Non-Productive Mixing: Preparation of Masterbatch

A Banbury-type tangential internal mixer, having a volume of 1200 cm^3^, was used for the preparation of the masterbatch. Particularly, two different phases were used. 

Phase 1: SBR, 642.96 g, was masticated at 40 °C for 30 s at 75 rpm. Then, 225.04 g of CRX 1391 was added, and the compound was mixed for 50 s at 40 °C and 75 rpm. Then, stearic acid, 12.86 g, CRX 1391, 160.74 g, oil, 102.87 g, and resins, 102.87 g, were added, and the compound was mixed for 1 min and 15 s at 40 °C and 75 rpm. The compound was then discharged.

Phase 2: Before starting the second phase, the compound was remixed at 40 °C for 30 s at 75 rpm. Then, Zinc salt, 6.2 g, Zinc Oxide, 12.39 g, 6PPD, 12.39 g, and CRX 1391, 30.98 g, were added and mixed for 1 min and 15 s at 40 °C and 75 rpm. Finally, the compound was discharged at 155 °C for 55 s at 75 rpm. A total of 1.309 Kg of the masterbatch was obtained.

The same masterbatch was used for both the composites. 

#### 2.4.2. Final Mixing: Addition of the Vulcanization Agents

The masterbatch was first mixed in a Brabender^®^-type internal mixer, for 1 min at 80 °C, 50 rpm. MBTS, thiuram, sulphur, and either DBTH or HMDP or poly(S-co-HMDP) copolymers as the crosslinking agents were added. Mixing was then carried out for 2 min at 80 °C, 50 rpm. The composites were discharged and cooled at room temperature and finally homogenized by being passed 5 times on the two-roll mill, operating at 50 °C, with the front and the back roll rotating at 30 rpm and 38 rpm, respectively, and 1 cm as the nip between the rolls.

### 2.5. Characterization Methods

#### 2.5.1. Characterization of HMDP

The instrument used for GC-MS analyses was an Agilent 5973 network mass selective detector (Agilent, Santa Clara, CA, USA) with a 6890 Series GC system mass spectrometer. The column used for all analyses was a J&W GC Column HP-5MS [(5%-phenyl)-methylpolysiloxane] 30 m, with a 0.25 mm internal diameter and a 0.25 μm film thickness.

ESI-Mass analyses were recorded with Electrospray ionization (ESI) with a Bruker Esquire 3000 plus ion-trap mass spectrometer instrument (Bruker, Billerica, MA, USA) equipped with an ESI Ion Trap LC/MSn System.

*NMR.* A Bruker 400 MHz instrument (100 MHz) was used for recording the ^1^H-NMR and ^13^C-NMR spectra of HMDP and poly(S-co-HMDP) copolymers. The temperature at which the spectra were recorded was 298 K, and 5 mg of PyC was dissolved in 1.0 mL of CDCl_3_ for ^1^H-NMR analysis. CDCl_3_: δ_H_ = 7.26 ppm was used as the reference chemical shift of the residual solvent. For ^13^C-NMR analysis, 40 mg of PyC was dissolved in CDCl_3_. Chemical shifts were reported in ppm with the solvent residual peak as the internal standard (CDCl_3_: δ_H_ = 77.16 ppm). 

#### 2.5.2. Characterization of Poly(S-co-PyC) Copolymers

The characterization methods for performing the elemental analysis, the differential scanning calorimetry, the gel permeation chromatography, and the infrared spectroscopy have been reported in a previous publication [47].

*NMR analysis.* 1D ^1^H-NMR and ^13^C-NMR spectra were recorded as reported above for HMDP. 2D ^1^H-^1^H COSY and ^1^H-^13^C HSQC were acquired with the same parameters that were already used for 1D experiments. The number of scans was adapted to the sample concentration to ensure a proper signal-to-noise ratio. 

#### 2.5.3. Characterization of Elastomeric Composites

*Crosslinking.* The crosslinking reaction of the rubber composites was performed at a temperature of 170 °C for a duration of 20 min. A sample of 5 g of crude rubber compound was placed into a Rubber Process Analyser (RPA) rheometer, from Alpha Technologies, Hudson, OH, USA. Initially, a strain sweep was performed at low deformation levels (0.1–25% strain) prior to the crosslinking stage. The sample was then maintained at 50 °C for 10 min and subjected to an additional strain sweep, also conducted at 50 °C. Then, the crosslinking process was carried out at 170 °C for 20 min, with an oscillation angle of 6.98% and a frequency of 1.7 Hz. During this process, the torque versus time curve was recorded, along with key parameters such as the minimum achievable torque (ML), the maximum achievable torque (MH), the time required to reach a torque equal to ML + 1 (tS1), and the time necessary to attain 90% of the maximum torque (T90).

*Analysis of the crosslink network.* Vulcanized composite samples were swollen in n-heptane for two days in a nitrogen atmosphere to allow the diffusion of reagents. The n-heptane was then removed, and the samples were washed with light petroleum and dried overnight at room temperature under reduced pressure. The samples were then immersed in toluene (200 mL) and placed in a glass tube flushed with nitrogen. The tube was then sealed and left in the dark for 72 h, the time required for equilibrium swelling to occur. After this time, the samples were dried by blotting with filter paper. Finally, the samples were quickly sealed in a clean container and weighed. The samples were then dried under vacuum at 70 °C for 24 h to remove the solvent and weighed again in the dried state. This is completed to obtain the amount of sorbed solvent and the weight of the dry network. The Flory–Rehner equation was applied to calculate the crosslinking network:

$$v_{e}=\frac{-[\ln\left(1-V_{r}\right)+V_{r}+X_{1}V_{r}^{2}]}{\frac{V_{1}\left(v_{r}^{1/3}-V_{r}\right)}{2}}$$

where *νe* is defined as the effective number of chains in a real network per unit volume; *χ*_1_ is the polymer–solvent interaction parameter, and V_1_ is the molecular volume of the solvent. V_r_ is defined as the volume fraction of polymer in a swollen network in equilibrium with pure solvent. The mathematical definition is reported in the following equation:

$$V_{r}=\frac{\frac{weight\;of\;dry\;rubber}{density\;of\;dry\;rubber}}{\frac{weight\;of\;dry\;rubber}{density\;of\;dry\;rubber}+\frac{weight\;of\;solvent\;absorbed\;by\;sample}{density\;of\;dry\;solvent}}$$

The procedure for measuring mono- and di-sulfidic crosslinks is very similar.

A total of 100 mg of crosslinked composite was placed in a 20 mL beaker with 100 mL of heptane. The sample was allowed to stand for 24 h. After this time, 3.8 mL of propanethiol and 4 mL of piperidine were added and left at room temperature for 2 h. The mixture was then washed 3 times in 50 mL of n-heptane and then filtered. The solid was left in n-heptane for another 24 h and then washed in light petroleum. The sample was filtered under vacuum and dried under reduced pressure for 24 h.

*Dynamic mechanical analysis in the shear mode.* In the RPA instrument reported above, curing was first performed. On the crosslinked samples, a first strain sweep was applied at 50 °C with a strain amplitude ranging from 0.1% to 25%. Then the instrument was set at the minimum strain amplitude (0.11), and the sample was subjected to these conditions to achieve a fully equilibrated system. Finally, the shear storage modulus G′, the shear loss modulus G″, and the ratio G″/G′ (tan δ) were detected and measured under strain-sweep oscillation, having a strain amplitude ranging from 0.1% to 25% and a frequency of 1 Hz.

*Dynamic mechanical analysis in the axial mode.* The rubber composite taken from the two-roll mill was rolled up to prepare a long cylinder. The cylinder was cutinton smaller cylinders and vulcanized to give cylindrical specimens with a length of about 25 mm and a diameter of about 12 mm. Dynamic mechanical measurements were performed with an Instron dynamic device in the traction-compression mode, at the following temperatures: 70 °C, 100 °C, and 120 °C, as reported in the literature [49]. An axial compressive pre-strain of 25% was applied on the specimen followed by a sinusoidal axial strain sweep having an amplitude of ±3.5% with respect to the applied pre-strain, with a 100 Hz frequency. The dynamic mechanical storage modulus (E′), the loss factor (E″), and the ratio of E″/E′ (tan δ) were registered.

*Tensile tests.* The composite from the two-roll mill was crosslinked in a vulcanizing press to produce a 1 mm thick vulcanized plate. ISO 37, the determination of the tensile properties of Vulcanized Rubber (ISO: Geneva, Switzerland, 2017), was used. Specimens were used for the tests. They were dumbbell-shaped, punched from the vulcanized plate, with a length of 10 ± 0.5 mm and a width of 4 ± 0.2 mm (type 3). On these specimens, the tensile tests were carried out with a Zwick universal testing machine (Zwick Roell Z001, Test expert, Ulm, Germany) and an optical extensometer. Stresses at 100, 200, and 300% elongation (σ_100_, σ_200_, σ_300_, respectively) as the stress at break (σ_B_), the elongation at break (ε_B_), and the energy at break were determined. Three measurements were performed for each sample.

*Test for the stability of the crosslinking network.* Curing was carried out in the RPA instrument reported above, at 170 °C for 10 min, with an oscillation angle of 6.98% and a frequency of 1.7 Hz. The temperature was then increased to 200 °C, in 6 min, without subjecting the sample to oscillation. When the system reached the temperature of 200 °C, the crosslinked sample was then held in the instrument at 200 °C for 20 min without oscillation.

## 3. Results and Discussion

### 3.1. Synthesis and Characterization of HMDP

**Figure 2:**
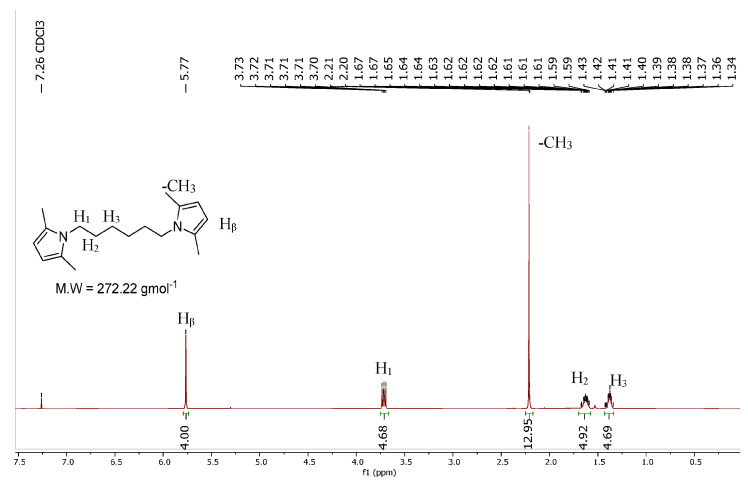
^1^H-NMR spectrum of HMDP.

HMDP was synthesized via a classical Paal–Knorr reaction. Hexamethylenediamine was first mixed in water and then heated up and mixed with 2,5-hexanedione. At the end of the reaction, the final crude product was only washed with distilled water due to the solubility of the two starting materials, in order to remove the unreacted substrates. The yield, 93%, was calculated by considering the final mmol of HMDP with respect to the starting mmols of hexamethylenediamine, the limiting reagent of the process. HMDP was obtained with a high Atom Economy and an almost null E factor. In Figure 2, the ^1^H-NMR spectrum of HMDP is reported.

### 3.2. Synthesis and Characterization of Poly(S-co-HMDP) Copolymers

The poly(S-co-HMDP) copolymers were obtained from the reaction of orthorhombic sulphur with the pyrrole derivative of hexamethylenediamine. HMDP was prepared as already reported [47], by dissolving the diamine in water, adding 2,5-hexanedione, and heating to 120 °C for 3 h, and it was obtained with a yield of up to 93%. The characterization was carried out by means of ^1^H-NMR and DSC and reproduced the results already reported [47]. The ^1^H-NMR spectrum is visible in Figure S1 in the Supporting Information. 

**Figure 3:**
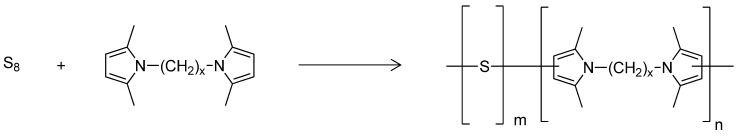
The synthesis of poly(s-co-HMDP) copolymer.

The scheme for the synthesis of poly(S-co-HMDP) copolymers is shown in Figure 3. 

The conditions reported in the seminal work on inverse vulcanization [35] were used. Two copolymers were used in this work as crosslinking agents, with different sulphur/HMDP ratios: 6 and 8.9. In this text, they are referred to as Copolymer 1 and Copolymer 2, respectively. The preparation of these copolymers was performed several times, to check the reproducibility of the process, and the same results were obtained. Residual sulphur and HMDP were not observed at the end of the reaction. The whole process was thus characterized by a high yield and, by neglecting the water released as the co-product of the synthesis of the pyrrole compound, by almost null E Factor, down to 0.11 [50].

The characterization confirmed their reproducibility. The preparation and characterization of the samples used in this work have been already reported [47]. For the sake of clarity, some of the copolymers synthesized in our previous work [47] were used as novel crosslinking agents in the present work. Particularly, the elemental analysis results reported in Table 2 and Table 3 and the gel permeation chromatography (GPC) results in Table 4 were the same as those already published in a scientific work [47]. Additionally, differential scanning calorimetry (DSC) and infrared (IR) analyses of the two copolymers are compared in the Supporting Information in Figures S1 and S2, respectively. In brief, evidence of pristine sulphur and HMDP was not found in the DSC traces. Additionally, the values of the glass transition temperatures (*T*_g_) of the poly(S-co-HMDP) copolymers were observed. In particular, a *T*_g_ value of 30 °C was observed for the copolymer with a molar ratio sulphur/HMDP = 6, and a *T*_g_ value of 22 °C was observed for the copolymer with a molar ratio sulphur/HMDP = 8.9.

#### 3.2.1. NMR Analysis

The NMR results, discussed in a previous publication [47], revealed that the sulphur essentially reacts with the β-position of the pyrrole ring. There were clear indications of a mono-substitution of the ring, and the di-substitution could not be excluded. In this work, the microstructures of Copolymer 1 and Copolymer 2 were carefully compared by means of ^1^H-, ^13^C-, 2D ^1^H-^1^H-COSY, and 2D ^1^H-^13^C HSQC NMR analyses. The different sulphur content in the copolymers is indeed a valuable tool to better elucidate the copolymer microstructure. For the sake of clarity, the attribution of the NMR peaks, also reported in ref. [47], is given in the text below. 

#### 3.2.2. ^1^H-NMR Analysis

**Figure 4:**
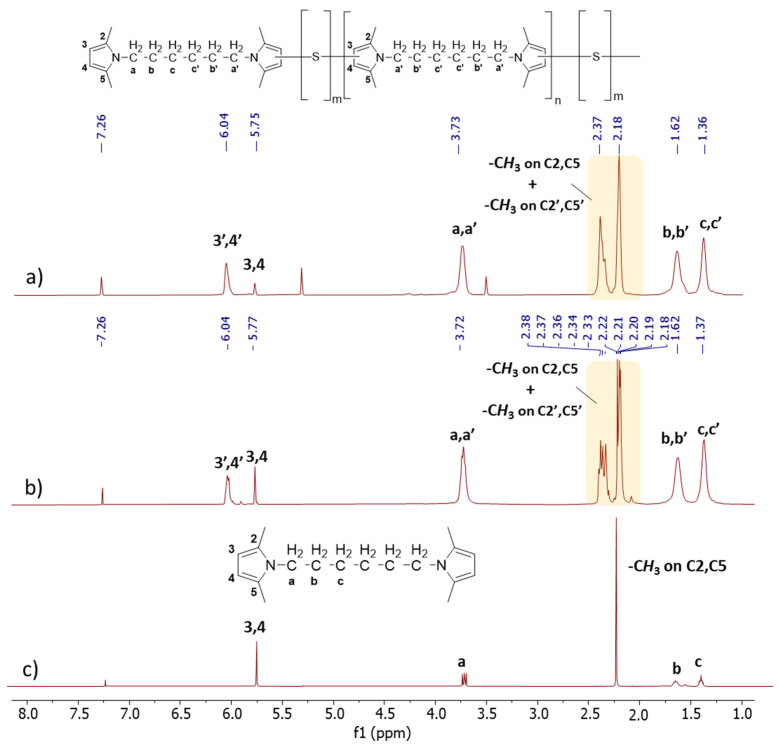
^1^H-NMR spectra (CDCl_3_, 400 MHz) of (**a**) copolymer 2, (**b**) copolymer 1, and (**c**) of HMDP.

*1D 1H-NMR*. In Figure 4, the 1D ^1^H-NMR spectra of Copolymer 1 (Figure 4a), Copolymer 2 (Figure 4b), and HMDP (Figure 4c) are shown.

The reference for the attribution of the peaks was the ppm value of the hydrogen atom signal (7.26 ppm). The assignments of HMDP and Copolymer 1 confirm what has already been reported [47]. In particular, Figure 4c shows the sharp singlets and defined multiplets of the HMDP spectrum. The spectra of the copolymers appear different: multiplets are present along with a new broad peak.

By examining the aromatic part of the copolymers’ spectra, the region between 5.5 and 6.7 ppm corresponding to heterocyclic aromatic protons, notable differences were observed between the spectra of Copolymer 1 and Copolymer 2. Specifically, variations were identified in the relative areas of the broad singlet at 5.8 ppm and the multiplet centred at 6.05 ppm. These signals were attributed [47] to the 3,4 protons in the β-positions of the pyrrole ring, without any covalent bonds with sulphur atoms, and to the 3′,4′ protons in the β-positions of the pyrrole ring containing sulphur atoms, respectively. On the basis of this assignment, it can be concluded that the amount of non-substituted pyrroles is lower in Copolymer 2, which indeed has a higher sulphur content.

In the spectrum of HMDP, the signals corresponding to the methyl groups on carbon atoms 2 and 5 are represented by a singlet at 2.21 ppm, and they are detected at a high field. In the spectra of the copolymers, this singlet transforms into a multiplet, with the respective signals detected in the spectre region between 2.17 ppm and 2.21 ppm. Additionally, a new set of peaks was found between 2.32 ppm and 2.37 ppm. In the spectrum of Copolymer 1, these signals can be attributed to the methyl groups on carbon atoms 2 and 5 of the pyrrole ring not substituted by sulphur atoms, the chain end, and to the methyl group on carbon atoms (2′ or 5′) next to a carbon atom in the β-position, which carries a sulphur atom (3′ or 4′). If the substitution with sulphur occurs at position 3′, the methyl group in position 5′ was detected with a higher field signal. On the other hand, the signal corresponding to the hydrogen atoms at positions 2 and 5 of the pyrrole ring is not detectable in the spectrum of Copolymer 2, probably due to the greater substitution with sulphur atoms. The signals corresponding to all the methylene units between the two pyrrole rings are at the same chemical shift as in the spectrum of HMDP but display greater complexity. This complexity may result from the overlap between the peaks arising from the methylene groups (a, b, c) adjacent to the pyrrole ring without sulphur atoms (the chain end) and the peaks associated with the methylene groups (a′, b′, c′) near the pyrrole ring containing sulphur atoms (in the repeating unit).

**Figure 5:**
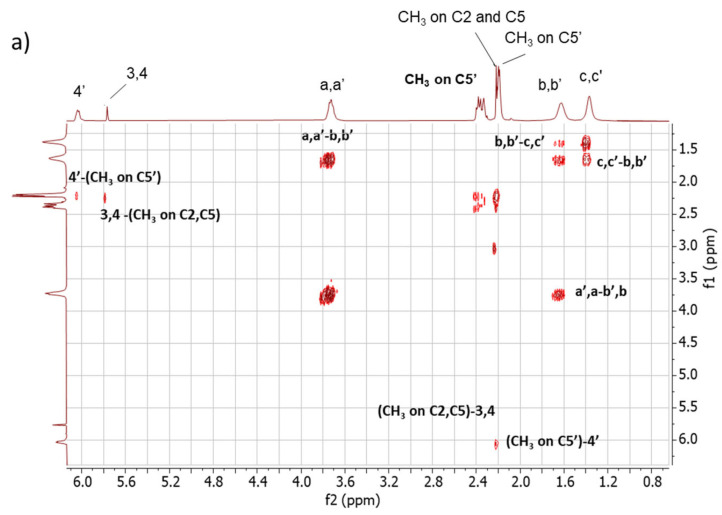

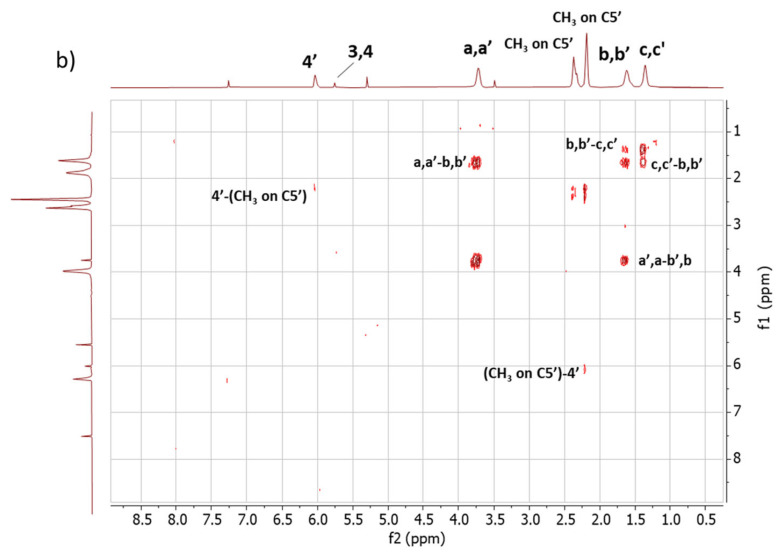
^1^H-^1^H COSY spectrum in CDCl_3_ of (**a**) Copolymer 1 and (**b**) Copolymer 2.

*2D ^1^H-^1^H-COSY NMR.* The assignments of the 1D ^1^H-NMR were further investigated by performing the COSY experiment. The ^1^H-^1^H COSY spectra of the copolymers are shown in Figure 5, Figure 5a for Copolymer 1 and Figure 5b for Copolymer 2.

The comparison between the COSY spectra of the two copolymers reveals remarkable differences. The correlations between 4′-(CH3 on C5′) and 3,4-(CH3 on C2, C5) were already commented on for Copolymer 1 [47] and are confirmed in this spectrum. In the spectrum of Copolymer 2, only the 4′-(CH3 on C5) correlation is detectable. These findings confirm what was observed by examining the 1D ^1^H-NMR spectra, i.e., the higher substitution with sulphur of the pyrrole ring in Copolymer 2.

#### 3.2.3. ^13^C-NMR Analysis

**Figure 6:**
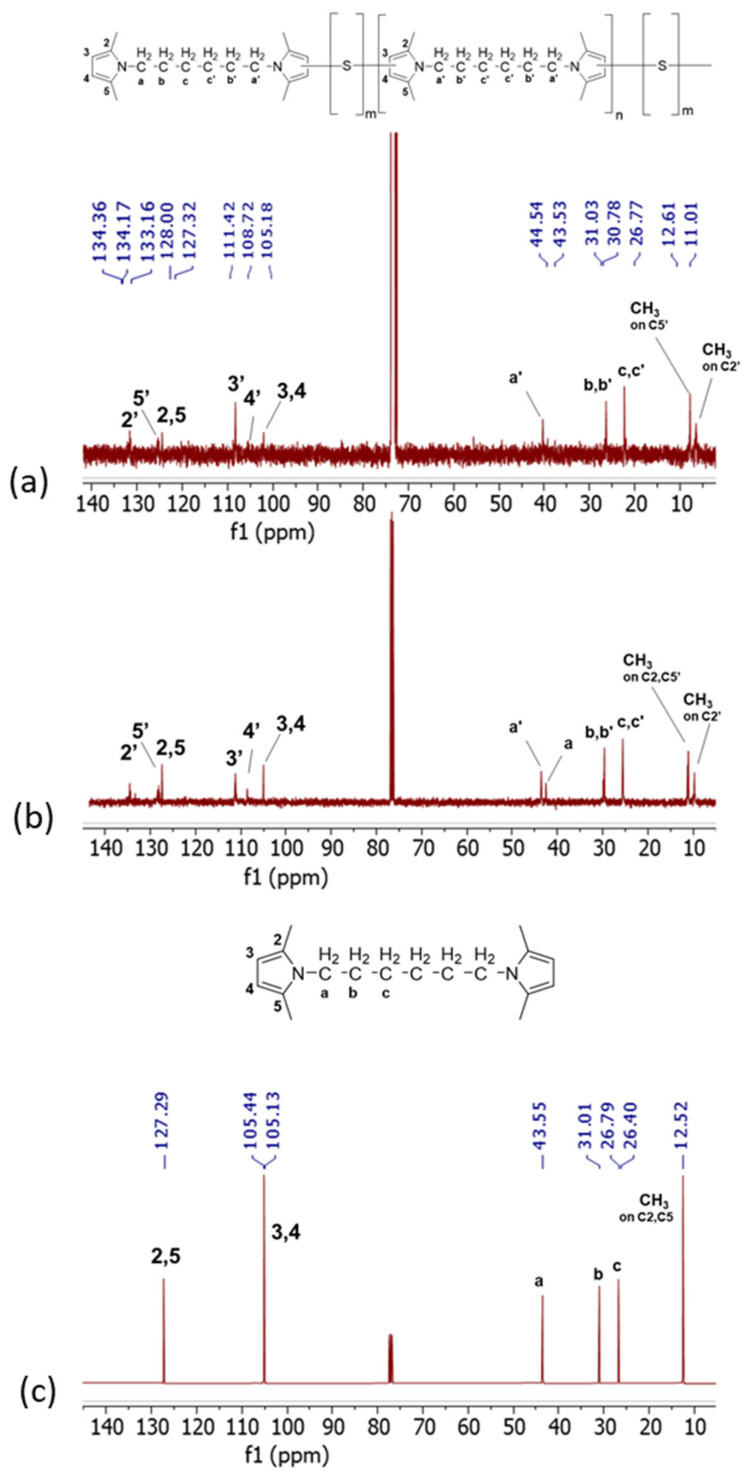
^13^C-NMR spectra (CDCl_3_, 100 MHz) of (**a**) Copolymer 2, (**b**) Copolymer 1, and (**c**) HMDP.

^13^C-NMR spectra are in Figure 6, for Copolymer 1 (Figure 6a), Copolymer 2 (Figure 6b), and HMDP (Figure 6c).

The C2, C5, C3, and C4 carbon atoms of pristine HMDP, and so of the non-substituted pyrrole ring, are attributed to the peaks at 127 ppm and at 105 ppm, respectively. New signals are visible in the ^13^C-NMR spectra of both copolymers. Particularly, C2′ at 134.17 ppm, C5′ at 127.13 ppm, C3′ at 111.23 ppm, and C4′ at 108.53 ppm are detected due to the interaction of the carbon atoms of the pyrrole ring with sulphur. The intensity of the peak due to C4′ is lower in the spectrum of Copolymer 2. The analysis of the spectrum of Copolymer 1 allowed us to hypothesize [47] that the carbon atoms of the pyrrole ring chemically react with sulphur atoms (3″ and 4″), which would decrease the intensity of the C4′ peak. Such intensity should be lower in the spectrum of a copolymer with a higher amount of sulphur, as observed in Figure 6a. The signals of 2″ and 5″ could fall in the region between 133 and 135 ppm. 

In the spectrum of Copolymer 1, new signals were also observed in the typical NMR region for aliphatic hydrocarbons, between 25 and 45 and between 10 and 13 ppm. In particular, the signals of the methyls, either in the unsubstituted pyrrole (C2 and C5) or in the substituted pyrroles (C2′ and C5′), are at 12.43, 12.24, and 10.82 ppm. Interestingly, in the spectrum of Copolymer 2, the signal due to the aliphatic carbon in the unsubstituted pyrrole is lower in intensity if compared with the same signal in the spectrum of Copolymer 1. This suggests what was already seen in ^1^H NMR for signals due to the end-chain carbons of the polymer.

**Figure 7:**
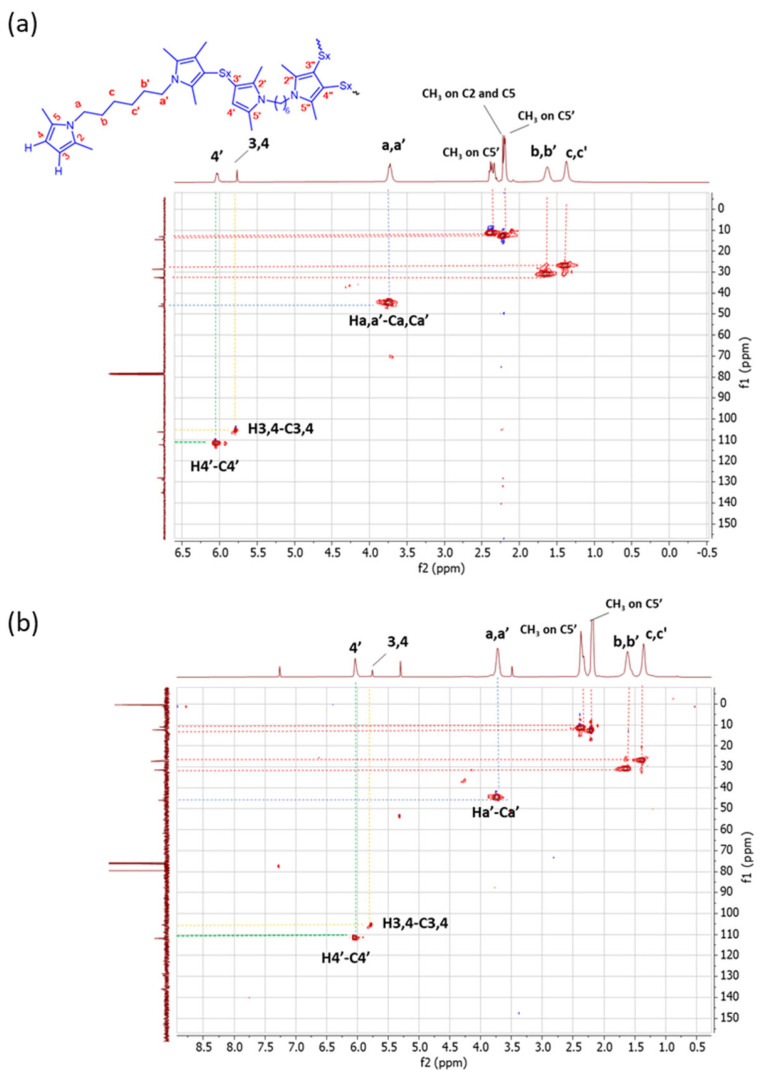
^1^H-^13^C HSQC *spectra* (in CDCl_3_) of (**a**) Copolymer 1 and (**b**) Copolymer 2.

*HSQC experiments.* HSQC experiments were performed in order to match the correlation between ^1^H-NMR and ^13^C-NMR. ^1^H-^13^C HSQC spectra are shown in Figure 7.

Different correlations in the HSQC spectrum of Copolymer 1 were detected. Particularly, the H4-C4 correlation and the H4′-C4′ correlation were observed. In the spectrum of Copolymer 2, only the H4′-C4′ correlation is remarkable. In addition, a correlation between H-a′ and Ca′ is revealed, and it is possible to distinguish between the C-b′ and C-c′ methylenes due to their related correlations with H-b′ and H-c′ in both spectra. These results confirm the higher substitution with sulphur of the pyrrole ring in Copolymer 2, in the β-position. 

The NMR findings indicate that the carbon atoms in the β-position of the pyrrole ring are the propagation site for the poly(S-co-HMDP) copolymer. The mono-substitution of the pyrrole ring seems to be largely favoured in Copolymer 1, whereas the substitution in both positions appears likely in Copolymer 2. The clear evidence that both the beta positions of the pyrrole ring can be attacked by sulphur is unprecedented and appears to be relevant for the development of pyrrole-based sulphur copolymers. It is worth adding that the reaction of sulphur and sulphur compounds with pyrrole rings has been documented, in the scientific literature, only by some of the authors [47,51] and indeed deserves further investigations. 

### 3.3. Preparation and Characterization of Elastomer Composites 

As mentioned in the Introduction, the objective of this work was to prepare an elastomer composite suitable for a high-performance tyre tread. The recipes are shown in Table 1. Typical ingredients were used: an SBR copolymer with a high styrene content, a high-surface-area carbon black, and a styrenic resin. These ingredients are suitable to enhance the hysteresis of the composite and hence the road grip of the tyre tread. A so-called efficient vulcanization system was used, with thiuram and a low sulphur/accelerator ratio, in order to reduce the length of the sulphur bridges between the polymer chains, thus enhancing the modulus of the composite and the stability of the crosslinking network. The elastomer composites were prepared via melt blending, by using an internal mixer and a traditional processing. The details can be found in Section 2.

Reference composites were prepared, with DBTH as the crosslinking agent to enhance the stability of the filler network as well as without any crosslinking agent. HMDP was used in the same molar amount as DBTH. The amount of poly(S-co-HMDP) copolymer was tuned to have the same HMDP content in the composite, and the amount of S_8_ was tuned to have the same amount of sulphur in all the composites. Two samples of poly(S-co-HMDP) copolymers were used, with different S/HMDP ratios in the chain, 6.0 and 8.9 (Copolymer 1 and Copolymer 2).

#### 3.3.1. Curing

The vulcanization reaction was carried out at 170 °C for 10 min. The values of the minimum modulus (M_L_), maximum modulus (M_H_), induction curing time (t_s1_), optimum curing time (t_90_), and curing rate are shown in Table 5. The vulcanization curves are shown in Figure S3 in the Supporting Information.

The addition of either DBTH or HMDP as the crosslinking agent did not appreciably affect the viscosity of the composites, as indicated by the M_L_ value. The slight increase in M_L_ observed for the composites from Entry 4 and Entry 5 could be due to the polymeric nature of the crosslinking agents. 

Higher values of M_H_ and (M_H_ − M_L_) were obtained in the presence of all the crosslinking agents, based either on DBTH or on HMDP. The value of M_H_ can be mainly attributed to the extent of the crosslinking network. However, the filler network does play a role, as the strain applied during the oscillation test is not sufficient to completely break it. As the filler content was the same in all the Entries, the comparison of the M_H_ values can give an indication of the extent of the crosslinking network, which can be thus assumed to be enhanced by the crosslinking agents. This induction was confirmed by the analysis of the crosslinking network, carried out with swelling measurements, discussed below in the text. 

The use of either HMDP or poly(HMDP-co-sulphur) copolymers led to a slightly lower induction curing time (lower t_s1_), whereas a lower optimum curing time (lower t_90_) and a higher curing rate were obtained with DBTH. It is useful to remember that t_s1_ and t_90_ are determined from the torque values of the composites. The faster crosslinking (lower t_s1_) observed in the presence of HMDP suggests that, with HMDP, crosslinking occurs via a radical-type mechanism that is known to occur in the first moments of a vulcanization [27]. DBTH seems to be more efficient when the vulcanization proceeds through the mechanism based on the active sulphurating species [27]. However, all the crosslinking agents lead to a more efficient vulcanization. It is worth commenting that the results obtained with pristine HMDP and with the copolymers, shown in Table 5, are in the same direction when compared with the reference compound. However, the differences are more pronounced with the copolymers. The poly(S-co-HMDP) copolymer is definitely more efficient as a crosslinking agent when its preparation is *ex-ante*. However, these results demonstrate that the HMDP can form the copolymer with sulphur in situ, during the preparation of the composite. The differences between the composites based on either Copolymer 1 or Copolymer 2 are slight. A higher sulphur content resulted in a lower optimum time of vulcanization and a higher curing rate.

#### 3.3.2. Crosslinking Network

The analysis of the crosslinking network was performed by means of swelling measurements, by applying the Flory–Rehner theory [52]. The data of the total X-linking density and of the relative amount of nominal mono/di and poly-sulphides are shown in Table 6. The analysis of the bridges only nominally refers to sulphur sequences, as the crosslinking agents introduce methylene groups between the elastomer chains. 

The crosslinking agents resulted in an increase in the total X-link concentration. These data appear to confirm the interpretations given above for the values of M_H_. A higher relative amount of long crosslink bridges was found for the composites with DBTH and with Copolymer 1, i.e., the copolymer with shorter sulphur bridges. A low amount of long crosslink bridges was found for the composites with HMDP and with Copolymer 2, i.e., the copolymer with longer sulphur bridges. Only preliminary comments and hypotheses can be made, in order not to overstretch conclusions without sufficient support from experimental data. The difference between HMDP and Copolymer 1 could be due to the preparation ex ante of the copolymer. The difference between Copolymer 1 and Copolymer 2 could be due to the different lengths of the sulphur bridges. The longer bridges in Copolymer 2 could react with the thiol used in the procedure for the determination of the short bridges, as described in detail in Section 2. However, it is indeed worth repeating that these are only hypotheses to be verified in future experiments. 

#### 3.3.3. Dynamic Mechanical Characterization in the Shear Mode

**Figure 8:**
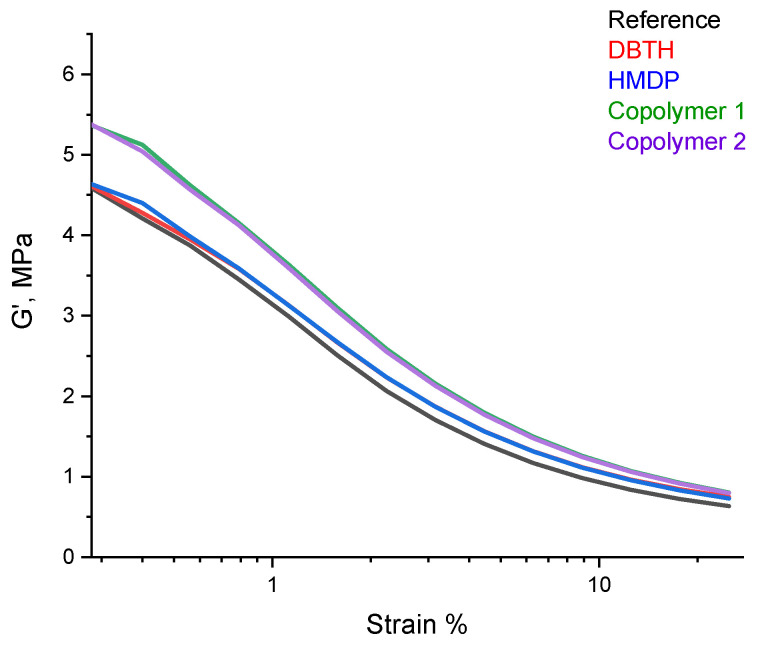
G′ vs. strain for the composites of Table 1.

A dynamic mechanical characterization in the shear mode was performed, by means of strain sweep tests, as described in detail in Section 2. The values of G′_γmin_, G′_γmax_, ΔG′, ΔG′/G′, G″_max_, and Tan δ (G″/G′) are shown in Table 7. The dependence of G′ on the strain amplitude is shown in Figure 8. 

Only preliminary comments can be made, as the experiments were not repeated a sufficient number of sufficient to allow a statistical analysis of the data. The values of G′ at the minimum strain were similar for the reference composite and for the composites with DBTH and HMDP. The poly(S-co-HMDP) copolymers prepared *ex-ante* gave rise to the highest values of G′ at the minimum strain. The values of G′ at the minimum strain give an indication of the extent of the filler network [53], which is unlikely to be remarkably affected by the crosslinking agents, used in a low amount. The values of G′ at the maximum strain are similar for all the composites, in particular for those containing a crosslinking agent. Hence, the composites with the crosslinking agents appear to have higher dynamic rigidity, and this is in line with the higher values of the crosslinking density, shown in Table 6. All the composites revealed, as expected, a reduction in the storage G′ modulus with the strain amplitude. The nonlinearity of the storage modulus is a phenomenon known as the Payne effect [54,55,56] and is due to the disruption of the filler network, which was above its percolation threshold in all the composites. The values of ΔG′/G′, a normalized index of the Payne effect, were similar for all the composites, and the values of Tanδ_max_ were lower for the composites with the crosslinking agent. These findings suggest that the higher value of G′ at the minimum strain for the composites with the poly(S-co-HMDP) copolymers could be due to the higher rigidity of these composites rather than to the poorer distribution of carbon black.

#### 3.3.4. Dynamic Mechanical Characterization in the Axial Mode

**Figure 9:**
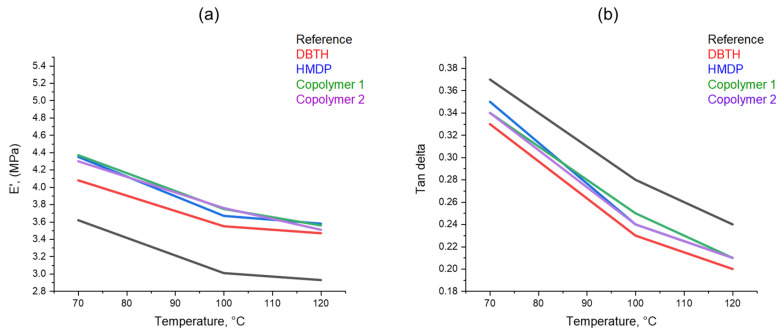
The dependence on temperature of (**a**) E′ and (**b**) Tan δ.

Dynamic mechanical properties from axial compression tests were determined as described in Section 2. The values of storage modulus E′, loss modulus E″, and tan δ (E″/E′) were obtained at 70 °C, 100 °C, and 120 °C. These temperatures were chosen in view of the target final application of elastomer composites, in high-performance tyre treads. The dependence on the temperature of E’ and tan δ is shown in Figure 9. The values of the parameters at all the temperatures can be seen in Table S1 in the Supporting Information. The composites with a crosslinking agent reveal higher dynamic rigidity and a lower tan delta than the reference composite. The highest values of the dynamic rigidity are shown by the composites with the poly(S-co-HMDP) copolymers. To explain the higher rigidity due to the poly(S-co-HMDP) copolymers prepared *ex-ante*, one could hypothesize the grafting of the copolymers on the CB surface, as recently reported [57] for this type of copolymers. These findings seem to be in line with the results shown above on the density of the crosslinking network. The reduction in the dynamic rigidity with the temperature is higher for the copolymers, compared with DBTH. This suggests that the structure of the copolymers could be improved, for example, by tuning the amount of sulphur. The comparison of the results obtained with HMDP and with poly(S-co-HMDP) copolymers confirms what was found with the curing tests and also with the strain sweep experiments: HMDP can form the copolymer with sulphur in situ, during the preparation of the composite.

#### 3.3.5. Tensile Tests 

**Figure 10:**
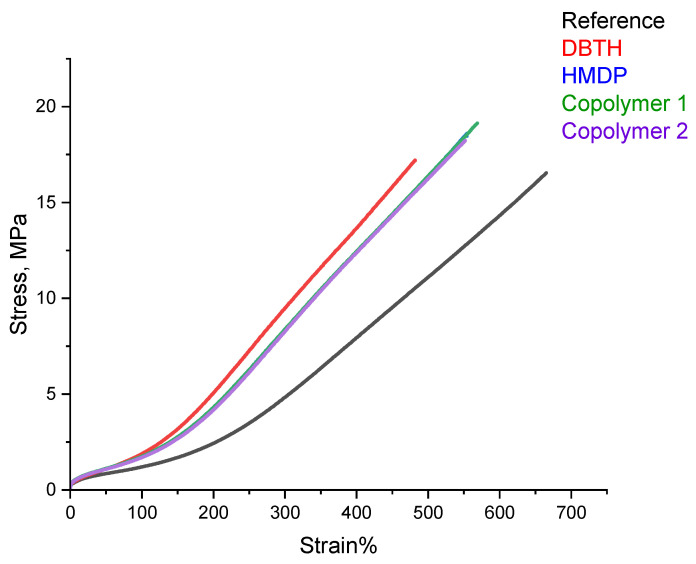
Stress vs. strain for the composites of Table 1.

Tensile tests were performed on the composites of Table 1, with quasi-static measurements. The stress—strain curves are shown in Figure 10, and the values of σ_100_, σ_300_, σ_B_, and ε_B_ are shown in Table S2 in the Supporting Information. 

All the composites with a crosslinking agent revealed higher values of stresses, at all the elongations and at break, and the lower elongation at break, compared with the reference composite. The composites with the HMDP-based crosslinking agents showed better ultimate properties, i.e., higher stress, elongation, and energy at break, compared to the composite with HMDP. This result really deserves attention. Indeed, the better ultimate properties occur in the presence of the higher dynamic rigidity commented on in the paragraphs above. It is unusual to have the simultaneous improvement of these properties. 

#### 3.3.6. Stability of the Crosslinking Network

To evaluate the stability of the crosslinking network, the cured composites were treated at high temperatures, as described in detail in Section 2. Curing was performed at 170 °C for 10 min, and then the composites were kept at 200 °C for 20 min, measuring the torque value. The values after the curing at 170 °C and after the treatment at 200 °C are shown in Table 8. The curves are shown in Figure S4 in the Supporting Information. 

This test is preliminary and gives only an indication of the stability of the crosslinking network. However, it can be observed that the poly(S-co-HMDP) copolymers led to a lower reduction in the modulus. To explain this finding, it could be hypothesized that the pyrrole rings favour the stability of the dynamic rigidity. In particular, the lowest reduction in torque was obtained with the poly(S-co-HMDP) copolymer with the lower sulphur content, Copolymer 1. It could be assumed that shorter and hence more stable sulphur bridges are formed with Copolymer 1.

## 4. Conclusions

This work demonstrates that circular materials can be upcycled for preparing elastomer composites for high-performance tyre treads. Poly(S-co-HMDP) copolymers were used as the crosslinking agents of elastomer composites. The copolymers were made with a problematic waste such as sulphur and with the pyrrole derivative of a chemical that could be prepared from the hydrolysis of an end-of-life polyamide. The process, from the synthesis of HMDP to the isolation of the copolymers, was characterized by the absence of solvents and catalysts. The synthetic pathway was characterized by a high yield and an almost null E Factor, particularly down to 0.11 when calculated by considering only the organic part involved in the synthesis of HMDP. Two poly(S-co-HMDP) copolymers with different sulphur contents were used as novel crosslinking agents in the preparation of rubber compounds and composites suitable for high-performance tyres, achieving more efficient curing, a higher density of the crosslinking network, higher dynamic rigidity, better ultimate tensile properties, and higher stability of the crosslinking network, when compared with a reference composite with a traditional vulcanization system, without any further crosslinking agent. Similar results were obtained with the poly(S-co-HMDP) copolymers and DBTH, the crosslinking agent that has been used for decades on a commercial scale. The copolymers resulted in a better balance of dynamic rigidity and ultimate tensile properties, allowing for an unusual simultaneous enhancement. The results were obtained with pristine HMDP, and the HMDP copolymers were in the same direction, but the differences with the reference composite were more evident with the copolymers, prepared *ex-ante*. However, these findings allow us to hypothesize that HMDP, due to its high reactivity with sulphur, can form copolymers with sulphur in situ, during the preparation of the composite. The structure of the sulphur copolymers is tuneable, by modifying the amount of sulphur and the structure of the organic spacer. Hence, these results potentially pave the way for families of crosslinking agents.

## Figures and Tables

**Table 1:** Recipes of the elastomer composites ^a^.

Entry	1	2	3	4	5
Crosslinking agent ^b^	=	DBTH	HMDP	Copolymer 1	Copolymer 2
SBR	100	100	100	100	100
CB CRX 1391	35	35	35	35	35
Stearic acid	2	2	2	2	2
CB CRX 1391	25	25	25	25	25
Oil	16	16	16	16	16
Resin	16	16	16	16	16
Zinc salt	1	1	1	1	1
ZnO	2	2	2	2	2
6PPD	2	2	2	2	2
CB CRX 1391	5	5	5	5	5
Sulphur	0.70	0.70	1.25	0.69	0.51
MBTS ^c^ 80%	2	2	2	2	2
Thiuram	0.6	0.6	0.6	0.6	0.6
DBTH	0.00	2.00	0.00	0.00	0.00
HMDP	0.00	0.00	0.79	0.00	0.00
Poly(HMDP-co-Sulphur)Ratio S/-(CH_2_)_6_ = 6	0.00	0.00	0.00	1.36	0.00
*HMDP*	*0.00*	*0.00*	*0.00*	*0.80*	*0.00*
*S_8_*	*0.00*	*0.00*	*0.00*	*0.56*	*0.00*
Poly(HMDP-co-Sulphur) Ratio S/-(CH_2_)_6_ = 8.9	0.00	0.00	0.00	0.00	1.53
*HMDP*	*0*	*0*	*0*	*0*	*0.79*
*S_8_*	*0*	*0*	*0*	*0*	*0.74*

^a^ The amounts are reported in phr; ^b^ for enhancing the stability of the crosslinking network; ^c^ dibenzothiazyl disulphide.

**Table 2:** Chemical composition of poly(S-co-HMDP) samples ^a,b^.

Sample	Carbon wt%	Hydrogen wt%	Nitrogen wt%	Sulphur wt%
Copolymer 1 ^c^	40.0	6.1	5.0	20.3
Copolymer 2 ^c^	49.8	7.9	6.0	23.2

^a^ Data from ref. [47]; ^b^ from elemental analysis; ^c^ the variation coefficient was between 0.4 and 0.5 for C, 4.9 and 5 for H, 1.7 and 2 for N, 2.2 and 2.5 for S, with the greater values for the polymer with the lower contents.

**Table 3:** The elemental analysis of poly(S-co-HMDP) copolymers used in the present work.

Entry ^a^	Carbon wt%	Standard Deviation	Hydrogen wt%	Standard Deviation	Nitrogen wt%	Standard Deviation	Sulphur wt%	Standard Deviation
1	40.0	±0.2	6.06	±0.3	4.96	±0.1	20.3	±0.5
3	49.8	±0.2	7.90	±0.3	6.05	±0.1	23.2	±0.5

^a^ See Table 1 of reference [47] for the molar ratio of sulphur and HMDP used in the synthesis of the copolymers.

**Table 4:** The weight average molecular weight (M_w_), the number average molecular weight (M_n_), and the dispersity (*Ð* = M_w_/M_n_) of Copolymer 1 and Copolymer 2. The results of the two copolymers have already been published in a previous work [47].

Sample	Molar Ratio Sulphur/Pyrrole Compound	M_w_ × 10^−3^	M_n_ × 10^−3^	*Ð* (M_w_/M_n_)
Copolymer 1	6.0	7.6	6.0	1.3
Copolymer 2	8.9	6.4	5.0	1.3

**Table 5:** Curing curve values derived from strain-sweep analysis.

Entry	1	2	3	4	5
Crosslinking agent	=	DBTH	HMDP	Copolymer 1	Copolymer 2
M_L_	2.90	2.76	2.81	3.12	3.10
M_H_	10.12	12.53	11.97	12.68	12.64
M_H_ − M_L_	7.22	9.77	9.16	9.56	9.54
t_s1_	2.69	2.76	2.56	2.44	2.45
t_90_	8.30	7.80	8.23	8.21	7.83
t_90_ − t_s1_	5.61	5.04	5.67	5.77	5.38
Curing rate ^a^	1.29	1.94	1.61	1.66	1.77
M_H_ *Relative values*	100	124	118	125	125

Curing rate ^a^ = M_H_ − M_L_/t_90_ − t_s1_.

**Table 6:** The crosslink density of the elastomer composite of examples 1–5.

Entry	1	2	3	4	5
Crosslinking agent	=	DBTH	HMDP	Copolymer 1	Copolymer 2
Total X-link mol/g × 10^−5^	1.16	1.87	1.67	1.73	1.94
Mono and di-sulphide (wt %)	97.10	89.80	97.60	87.00	94.50
“Poly-sulphide” (wt %)	2.90	10.20	2.40	13.00	5.50

**Table 7:** The values of G′, ΔG′, G″, and tan δ from a strain-sweep analysis.

Entry	1	2	3	4	5
	Reference	DBTH	HMDP	Copolymer 1	Copolymer 2
G′_γmin (0.2%)_	4.58	4.60	4.63	5.36	5.37
G′_γmax (25%)_	0.63	0.75	0.73	0.80	0.79
ΔG′	3.95	3.85	3.90	4.56	4.58
ΔG′/G′	0.86	0.84	0.84	0.85	0.85
G″_(max)_	0.77	0.71	0.76	0.84	0.83
Tan δ _(max)_	0.37	0.33	0.35	0.35	0.35

**Table 8:** Curing curve values derived from the strain-sweep analysis of elastomer composites of Table 1.

Entry	1	2	3	4	5
Crosslinking agent	=	DBTH	HMDP	Copolymer 1	Copolymer 2
S’_max_^a^	10.5	13.1	12.5	12.8	12.9
S’_end_^b^	8.9	11.2	10.6	11.3	11.2
$100\times \frac{S^{\prime }\max-S^{\prime }end}{S^{\prime }max}$	15.2	14.5	15.2	11.7	13.2

^a^ Maximum value of torque, achieved after the curing at 170 °C; ^b^ value of torque measured after the treatment at 200 °C.

## Data Availability

The data that support the findings of this study are available from the corresponding author upon reasonable request.

## Supplementary Materials

The following supporting information can be downloaded at: https://www.mdpi.com/article/10.3390/polym16192802/s1, Figure S1: DSC curves (second heating) of Copolymer 1 (black line) and Copolymer 2 (blue line); Figure S2: FT-IR spectra of (**a**) pristine HMDP, (**b**) poly(S-co-HMDP) material having molar ratio Sulphur/HMDP equal to 6 and (black line) and (**c**) poly(S-co-HMDP) material having molar ratio Sulphur/HMDP equal to 8.9 (blue line); Figure S3: Curing curves for the composites of Table 1. Curing at 170 °C for 20 min; Figure S4: Stability test curves for the composites of Table 1. Curing reaction was performed at 170 °C for 10 min and, subsequently, at 200 °C for 20 min. Table S1: Dynamic mechanical properties from axial measurements of composites in Table 1; Table S2: Tensile properties of composites in Table 1.
