# Supplementary material for: Sulphur Copolymers with Pyrrole Compounds as Crosslinking Agents of Elastomer Composites for High-Performance Tyres
Source: Polymers (Basel). 2024 Oct 3;16(19):2802. doi: 10.3390/polym16192802 (PMC11478510; doi:10.3390/polym16192802)
Supplement: Supplementary file 1 [file polymers-16-02802-s001.zip › polymers-3158898-supplementary.pdf]

Supporting Information

Electronic Supplementary Information (ESI)

Sulfur copolymers with pyrrole compounds as crosslinking agents of elastomer composites for high performance tyres

Simone Naddeo, Vincenzina Barbera and Maurizio Galimberti*

Politecnico di Milano, Department of Chemistry, Materials and Chemical Engineering "G. Natta", Via Mancinelli 7, 20131 Milano, Italy

*Corresponding authors. E-mail: maurizio.galimberti@polimi.it (Maurizio Galimberti)

Characterization of poly(S-co-HMDP) copolymers

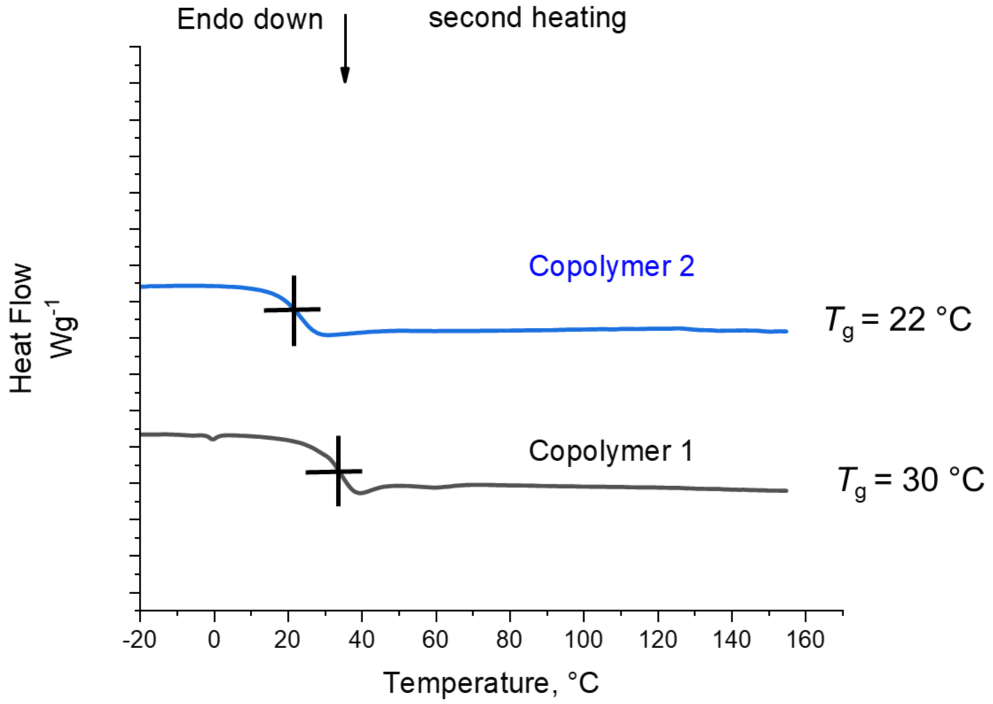

Figure S1: DSC curves (second heating) of Copolymer 1 (black line) and copolymer 2 (blue line)

FT-IR characterization of copolymer 1 and copolymer 2

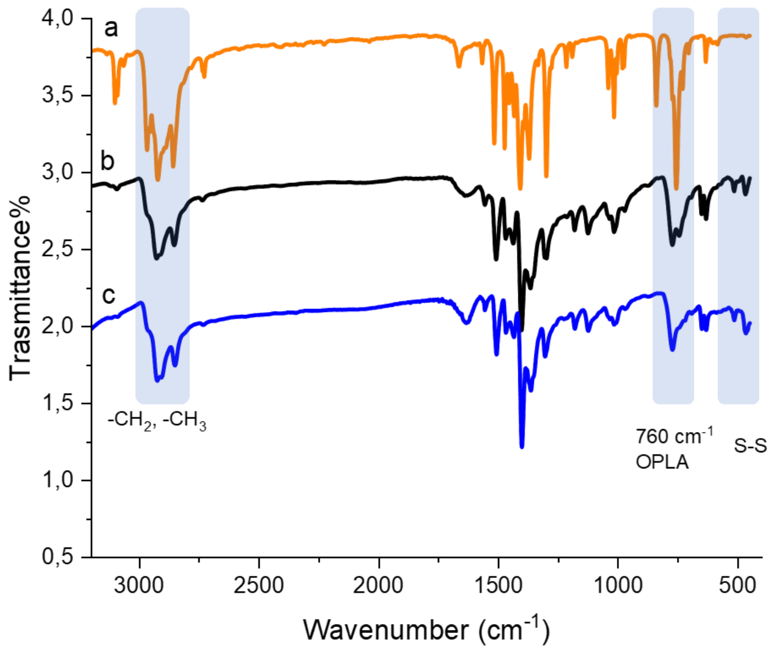

Figure S2. FT-IR spectra of (a) pristine HMDP, (b) poly(S-co-HMDP) material having molar ratio Sulfur/HMDP equal to 6 and (black line) and (c) poly(S-co-HMDP) material having molar ratio Sulfur/HMDP equal to 8.9 (blu line)

Characterization of the composites of Table 1

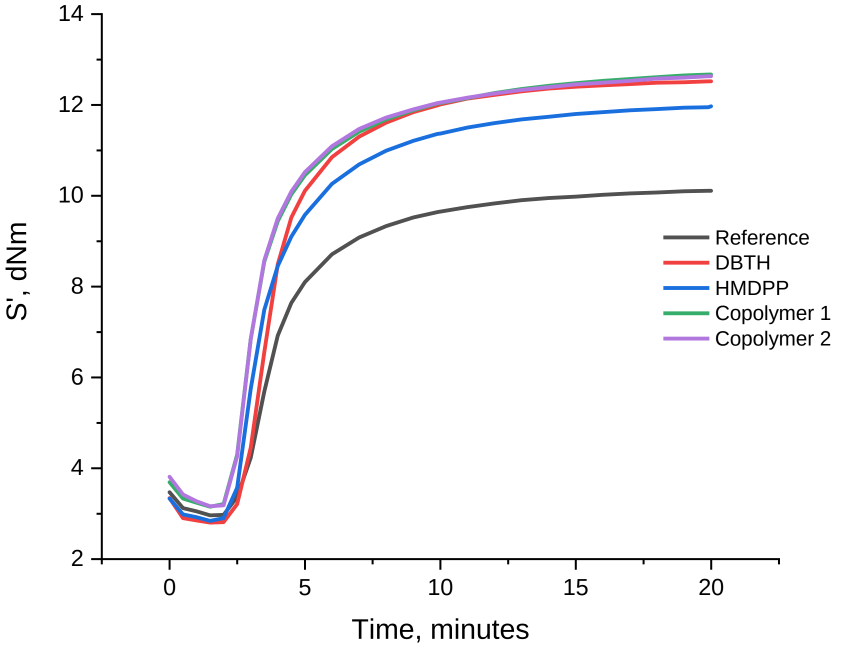

Figure S 3. Curing curves for the composites of Table 1. Curing at 170°C for 20 minutes

Table S1. Dynamic mechanical properties from axial measurements of composites in Table 1

Entry		1	2	3	4	5
Crosslinking agent		=	DBTH	HMDP	Copolymer 1	Copolymer 2
	Temperature (°C)					
E' (MPa)	70	3.62	4.08	4.35	4.37	4.30
	100	3.01	3.55	3.67	3.75	3.76
	120	2.93	3.47	3.58	3.56	3.51
E'' (MPa)	70	1.33	1.36	1.51	1.49	1.45
	100	0.84	0.83	0.90	0.94	0.92
	120	0.71	0.69	0.76	0.74	0.73
Tan Delta	70	0.37	0.33	0.35	0.34	0.34
	100	0.28	0.23	0.24	0.25	0.24
	120	0.24	0.20	0.21	0.21	0.21
**Δ**E' (E'@70°C-E'@120°C) (MPa)		0.69	0.61	0.77	0.81	0.79

Table S2: Tensile properties of composites in Table 1**.**

Entry	1	2	3	4	5
Crosslinking agent	=	DBTH	HMDP	Copolymer 1	Copolymer 2
σ_{100} (MPa)	1.25	1.89	1.73	1.72	1.70
σ_{300} (MPa)	5.17	9.47	8.17	8.29	8.40
σ_B (MPa)	16.54	17.34	18.46	18.69	18.31
ε_B (%)	665.61	482.33	554.73	569.17	552.20
Energy at break (MJ/m^3)	44.71	36.74	44.49	47.33	44.11

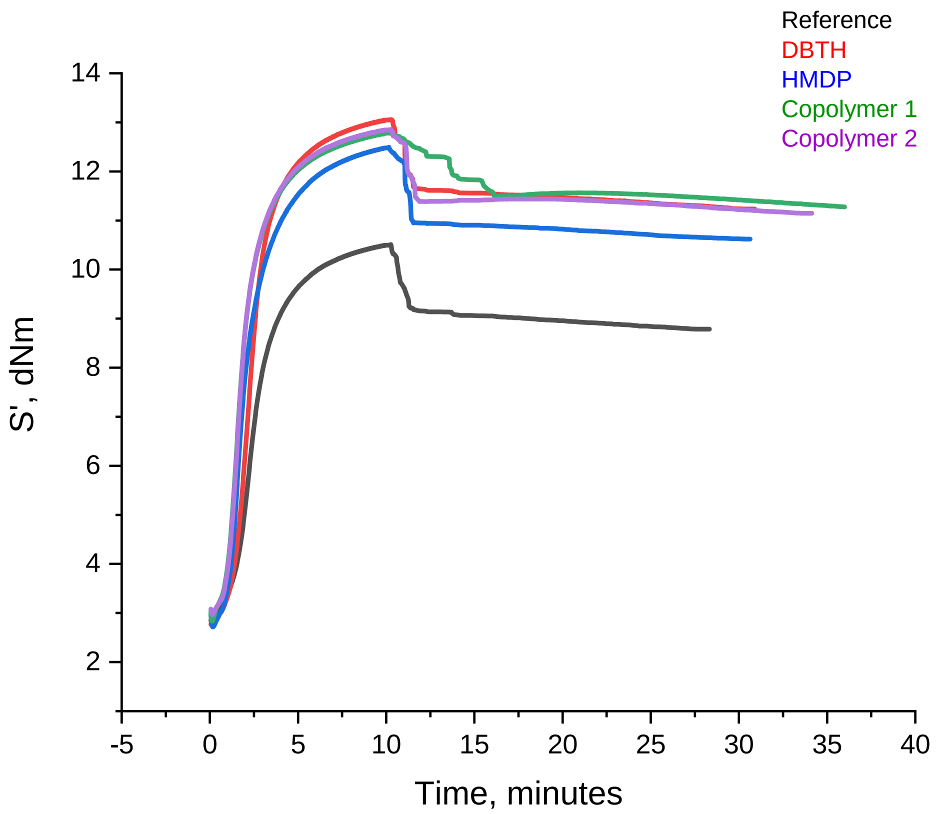

Figure S4. Stability test curves for the composites of Table 1. Curing reaction was performed at 170°C for 10 minutes and, subsequently, at 200°C for 20 minutes.
